# Body-Centered Interventions for Psychopathological Conditions: A Review

**DOI:** 10.3389/fpsyg.2019.02907

**Published:** 2020-01-24

**Authors:** Mary S. Tarsha, Sohee Park, Suzi Tortora

**Affiliations:** ^1^Department of Psychology, Kroc Institute for International Peace Studies, University of Notre Dame, Notre Dame, IN, United States; ^2^Department of Psychology, Vanderbilt University, Nashville, TN, United States; ^3^Dancing Dialogue: Healing and Expressive Arts, New York, NY, United States

**Keywords:** body-centered, interventions, taxonomy, brain-body communication, therapies alternatives

## Abstract

World-wide, billions of dollars are spent each year on body-centered interventions to alleviate both physical and psychological pathologies. Given the high demand and increasing popularity of body-centered interventions, there is need for a systematic organization of empirical evidence associated with body-centered therapies. This article reviews the psychological effects of body-centered interventions on emotional well-being, including both self and other-administered (receptive) therapies. Theory behind body-centered interventions rely upon the bidirectional communication pathway between the brain and body. We investigated the bidirectional communication pathway between the brain and body by evaluating evidence across multiple body-centered therapies. The research reviewed includes studies that investigate effects of massage therapy, reflexology, acupuncture, functional relaxation, emotional freedom technique, Rolfing, yoga, tai-chi, and dance/movement therapy on psychological conditions across the lifespan. Results demonstrated that overall, massage therapy, tai-chi, dance/movement therapy, functional relaxation, reflexology, acupuncture and emotional freedom technique seem to alleviate stress, depression, anxiety, bipolar disorder and facilitate pain reduction. Of these, the most robust evidence available was for massage therapy, indicating it is an effective intervention for numerous age groups and populations. Rolfing and reflexology had the least amount of support, with few studies available that had small sample sizes. Although these conclusions are limited by scarcity of high-quality empirical data and contradictory findings, available evidence indicates that body-centered interventions can be effective in reducing psychopathology and supports the proposed mechanism of the bidirectional pathway between the brain and body: the body holds the potential to influence the mind. Integrating body-centered therapies in both clinical settings and as self-care could lead to better outcomes. Lastly, we propose the first taxonomy of body-centered interventions and empirical evidence of their effectiveness for clinicians and researchers.

## Introduction

Body-centered therapies and interventions have always been an integral part of traditional medical systems. Restoration of the balance between bodily humors and Nature were important to Hippocrates (460-377 B.C.) and ancient Greek physicians who believed in the healing effect of laying-on-of-hands and the importance of touch (Calvert, 2002). Later in Rome, Asclepiades promoted naturalistic therapeutic methods such as a healthy diet, massage and physical exercise. Healing through a holistic approach of integrating mind and body was also emphasized in the Traditional Chinese Medicine (Abbott and Lavretsky, 2013), Ayurveda (Mishra et al., 2001), and Traditional Arabic and Islamic Medicine (Al-Rawi and Fetters, 2012).

Much of the developing world continues to rely on traditional medical practices (World Health Organization, 2013, 2019), and in Western countries, a majority of the population has utilized some form of complementary medicine including body-centered therapies in lieu of or in conjunction with conventional biomedical interventions for both emotional and physical relief (Tracy et al., 2005). The 2007 National Health Interview Survey found that Americans spent $33.9 billion in the previous year on complementary or alternative medicine practices which include numerous body-centered interventions such as yoga, tai-chi, Qi Gong and relaxation techniques (Barnes et al., 2009). On massage therapy alone, Americans spent more than $14.2 billion and in total, $11.9 billion was spent on complementary practitioner visits, which amounts to one-quarter of total out-of-pocket spending on physician visits for the year. Given the high demand and increasing popularity of body-centered interventions, there is need for a systematic organization of empirical evidence associated with body-centered therapies (Röhricht, 2009). This review synthesizes the empirical evidence of major body-centered interventions and their effectiveness on psychological conditions and mental health, crafting a taxonomy of these heterogeneous somatic interventions.

**FIGURE 1:**
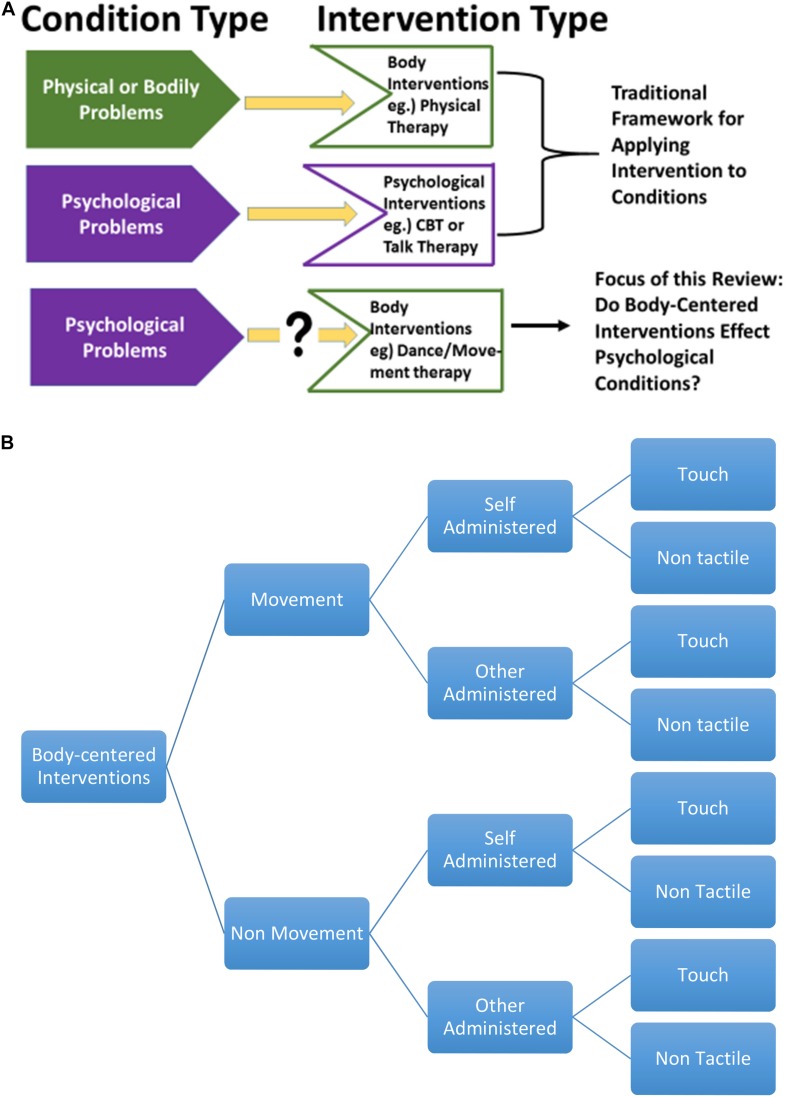
**(A)** Mind-body targeted interventions. **(B)** Classification of psychological and body-centered interventions.

The central nervous system’s control of the body and somatic functioning forms the basis for understanding the etiology and treatment of diseases in the modern biomedical system. Typical treatment of a disease, after diagnosis, entails matching the condition with the appropriate therapy or intervention. A particular psychological condition is most likely treated with a psychological therapy while a physical ailment involves direct intervention of the body (see Figure 1A). Research and practice of clinical psychology and psychiatry tend to focus on the impact of the brain/mind on mental states (e.g., Cognitive Behavioral Therapy for depression). The impact of changing cognitive framework on the body (e.g., Cognitive Behavioral Therapy to reduce stress response or chronic pain) has also been studied extensively (Cherkin et al., 2016). However, the effects of body-centered therapies on mental states have not been extensively studied even though the benefits of physical exercise on neuroplasticity, cognition and mood have been demonstrated (Salmon, 2001; Hötting and Röder, 2013). To summarize, the literature on the role of the brain/mind-based therapies on psychological disorders is robust. The literature on the role of the brain/mind-based therapies on the body is also quite robust. However, the literature on the role of body-centered therapies on psychological disorders is not well defined; this is the focus of our review.

Most of the abovementioned therapies assume that the relationship between the body and the brain is unidirectional—the brain controls and influences the body. However, there is growing evidence that there exists a communication pathway between the brain and body that is not limited to one direction but rather, is bidirectional; the brain not only influences and controls the body but the body communicates to the brain (Lutz et al., 2007). One theory behind body-centered interventions relies upon this bidirectional relationship between the brain and body suggesting that through the body one can alter or influence the brain and emotions yielding positive health benefits. These bidirectional communications (i.e., from the brain to the body, and from the body to the brain) are thought to takes place via three routes: the autonomic nervous system, the endocrine system and the immune system (Lutz et al., 2007). In this review, we aimed to expand our understanding of the bidirectional communication pathway between the brain and the body by focusing on the crucial influence of the body on the brain and presenting evidence across multiple body-centered interventions. With this goal, we investigated the effectiveness of body-centered, somatic interventions upon psychopathology.

To accomplish this goal, we developed a taxonomy of body-centered interventions and evaluate existing empirical evidence for each of them regarding *psychological* benefits. We organized each therapy according to three different factors: movement, method, and tactile involvement. The first factor of utilization of movement (or lack of movement) is important for physicians and patients who are seeking a body-centered therapy but cannot generate somatic movements. In this way, distinctions between movement and non-movement therapies is critical. Next, each body-centered intervention was further classified according to the administration method: delivered by another person or through self-administration. This categorization was employed because there are different known neurological effects for active versus passive movements (Shimada et al., 2010). Finally, each body-centered intervention was grouped according to tactile involvement—the therapy either utilizes touch or is non-tactile. Again, this categorization is important because (as we discuss below) traumatic events hold the potential to create touch-aversion (Rothschild, 2014). Lastly, for each body-centered intervention, definitions, quality of available empirical evidence, and proposed or known mechanisms were expounded (see Figure 1B).

Literature searches and the procuring of research articles were conducted with PubMed, Web of Science and Google Scholar. Priority was given to meta-analyses and Cochrane reviews above individual empirical studies.

## Non-Movement Therapies

### Massage Therapy: Tactile, Other-Administered

Massage therapy is the manual manipulation of soft tissue for healing or well-being (Moyer et al., 2004) and has been shown to alleviate a variety of symptoms across the lifespan from premature infants to the elderly (Field, 2014; Nakano et al., 2019). Its effectiveness has been demonstrated for migraines, depression, asthma, low-birth weight in infants, general pain reduction, fibromyalgia, high blood pressure, stress reduction, and overall increase in well-being and immune functioning (Cady and Jones, 1997; Field, 1998; Moyer et al., 2004; Ernst et al., 2007; Russell et al., 2008; Lee et al., 2010; Li et al., 2014). Additionally, it is effective at reducing pain in cases of physical injury or surgery (Diego et al., 2002; Hernandez-Reif et al., 2005; Bauer et al., 2010; Cutshall et al., 2010), and enhancing immunological function with the added benefit of simultaneously reducing negative emotional symptomology such as anxiety, depression and anger (Cassileth and Vickers, 2004; Hernandez-Reif et al., 2004; Kutner et al., 2008; Ernst, 2009). Thus, massage therapy carries a twofold physical and emotional benefit; it alleviates not only somatic symptomology but enhances psychological well-being.

The twofold physical and emotional pain alleviating attribute of massage therapy makes sense in light of the underlying circuitry involved in the brain’s representation of pain. Neuroimaging reveals that the circuitry involved in induction of both emotional pain, such as social rejection, and physical pain evoke activation of similar neurological regions (Eisenberger and Lieberman, 2004). In both situations, the dorsal subdivision of the anterior cingulate cortex, areas 240 and 320, and right ventral prefrontal cortex are activated (Eisenberger et al., 2003). Neurologically, both social/emotional and physical pain involve similar neural processes, suggesting that the activation of pain, rather than the source, is the key. In the brain, pain is recognized as pain regardless of its source, be it emotional or physical. However, only social pain involves the reliving of this pain each time memory is recalled (Chen et al., 2008; Jin and Josephs, 2016). This physical-social pain overlap in the brain becomes important when evaluating the effectiveness and mechanisms of massage therapy. Due to the neurological overlap in the brain in perceiving both emotional and physical pain, massage therapy’s twofold effectiveness at relieving physical pain and enhancing emotional well-being is expected.

#### Massage Therapy for Infants and Children

The benefits of massage therapy are not limited to the adult population but extend to both children and elderly persons. Massage therapy for infants has generated much research in the previous three decades demonstrating a significant decrease in physical and emotional stress. Following intervention, preterm infants demonstrate an increase in insulin and insulin-like growth factor 1 (Field et al., 2008), increase in body temperature (Diego et al., 2008), alleviation of sleep problems (Field and Hernandez-Reif, 2001; Mindell et al., 2018), and reduction in stress by means of increased vagal tone (Field et al., 2010a) and increase in triglycerides, an important benefit for premature infants (Field et al., 1996, 2010b; Field, 2019). Due to the number of benefits and its effects on physical and emotional states, massage could be utilized to facilitate emotional regulation in infants.

For older children, massage therapy has been used as either a supplemental or alternative treatment for asthma, autism and ADHD. Both children with ADHD and autism benefited from massage therapy and exhibit a decrease in anxiety and cortisol levels and increase in overall positive mood and attention; also, in both groups, teachers and parents report better communication and task-oriented attention (Field et al., 1998; Escalona et al., 2001; Khilnani et al., 2003). Other studies show that asthmatic children demonstrate an immediate decrease in cortisol, behavioral anxiety and increase in pulmonary functioning following massage therapy (Field, 1998; Moyer et al., 2004; Abdel Fattah and Hamdy, 2011).

A handful of studies that have investigated the benefits of massage therapy on elderly and found that hand massage therapy immediately and significantly reduced aggressiveness in nursing home residents compared to music therapy alone (Remington, 2002). Massage therapy also significantly reduced anxiety and depression and increased well-being, general health and perceived stress compared to guided relaxation (Sharpe et al., 2007).

#### Mechanisms

Possible mechanisms by which massage therapy induces these emotional and biophysiological changes have been proposed. Some suggest that pressure applied during massage stimulates parasympathetic activity and/or increases vagal tone (Diego et al., 2004; Moyer et al., 2004). Increasing vagus nerve stimulation, also known as the tenth Cranial Nerve, and consequently reducing symptoms of psychopathology aligns with previous research investigating respiratory sinus arrhythmia (RSA), the online biomarker of parasympathetic functioning (Beauchaine, 2015). According to the Polyvagal theory (Porges, 1985, 1995, 2007, 2011), the vagus nerve (measured via RSA functioning) serves as the critical mechanism of attention, emotion, self-regulation, communication, social behavior and adaptability. Consequently, the vagus nerve is operative in multiple psychopathological conditions (Beauchaine and Thayer, 2015). The proposed mechanism is that massage increases vagal functioning which is a means of increasing parasympathetic function and ameliorating symptoms associated with dysregulated autonomic activity.

Field (2010) suggested that the pain-relieving property of massage therapy is attributed to a reduction in substance P and increase in serotonin and highlights that the overall benefits of positive touch include specific physiological changes. Others suggest that possible changes in body chemistry, such as the release of the endorphin serotonin into the bloodstream, ameliorates pain and provides feelings of relief or well-being (Andersson and Lundeberg, 1995).

Price (2007) argues that in adults, the therapeutic recovery process is intrinsically related to the integration of the self, specifically the integration of sensory and emotional experience. She argues that as a protective mechanism, victims of abuse (i.e., sexual abuse) dissociate these aspects of the self, derailing healthy functioning in adulthood. Consequently, massage therapy is used as a means of integrating the individual’s emotional and somatic experiences, catalyzing greater awareness of both phenomena. Field et al. (1997) demonstrated that massage therapy mitigates the effects of sexual abuse such as decreasing cortisol, depression, and anxiety. At the end of 1 month receiving massages, women with a history of sexual abuse exhibited a 31% decrease in urine cortisol levels, even in the midst of on-going life stresses.

Whereas more research is needed to investigate the emotional and somatic integration of massage therapy and possible mechanisms, there is growing evidence that massage therapy does increase serotonin and decrease cortisol yielding an alleviation of negative emotional symptomology such as depression, anger and enhancement of positive mood (Field et al., 2005; Field, 2010). However, more research is necessary to establish the dosage (or amount) and type (hand, foot, etc.) of massage therapy that is needed for each population and/or condition. In addition, most massage studies utilize cross-sectional rather than longitudinal designs and consequently, the durations of massage therapies effects are not established. Further, if massage therapy is to be implemented as a supplementary therapy, that is, a therapy that is given in addition to typical biomedical practices, more research is needed to understand possible interactions between massage therapy and other interventions such as pharmacological agents, physical and nutritional therapies.

### Functional Relaxation Therapy: Non-tactile, Self-Administered

Relaxation therapy is defined as a pedagogical approach toward inducing a reduction of tension without using external resources, utilizing techniques such as holding a representation, image or word in one’s mind, contracting and relaxing specific muscles, breathing and sometimes, small movements or posture changes (van Dixhoorn and White, 2005). Typically taught in an intense period spanning several months, relaxation therapy can be taught individually or in a group and is often referenced as stress management, psychological or nursing intervention and to some, relaxation therapy is synonymous with meditation. However, contrary to typical meditative practices, relaxation therapy emphasizes awareness of low and high tension in daily life and coaches individuals in how to unwind from stressful episodes.

Across studies, there is a lack of precision regarding functional relaxation therapy and how it differentiates from other therapies. In addition, for those studies using functional relaxation therapy and integrating breathing techniques, the alteration of RSA needs be taken into consideration, in addition to other pathways, as the neurobiological mechanism by which symptoms are reduced.

This body-oriented psychologic therapy has been found to be effective in reducing tension headaches, non-cardiac (non-specific) chest pain, psychosomatically influenced asthmatic diseases and irritable bowel disease (Loew et al., 2000, 2001; Lahmann et al., 2008a, b, 2009). In addition to alleviating physiological symptoms, functional relaxation therapy significantly reduced anxiety and stress, both chronic and circumstantial, in the above-mentioned studies. However, more precision regarding functional relaxation therapy is needed and more random controlled trials with larger samples are needed to further investigate its effectiveness on psychological conditions.

### Acupuncture: Tactile, Other-Administered

A healing modality utilized for more than 2,000 years, acupuncture is considered a premier medical skill in Eastern Asian medicine and has gained popularity in western countries in the last few decades with an estimated 3 million Americans a year pursuing acupuncture treatment (Bai and Lao, 2013). The methodology behind acupuncture includes the insertion of fine needles into an individual in specified, defined areas with the aim of relieving pain. There is evidence from animal models that demonstrate that acupuncture evokes the release of different neuropeptides, such as the analgesic adenosine, into the central nervous system. Experimental studies show that 50–70% of patients report a decrease in chronic pain after short-term acupuncture treatment (Murray, 1995). Acupuncture has been effective at reducing tension headaches/migraines, Parkinson’s disease symptoms, fibromyalgia, chronic neck and back pain, osteoarthritis pain and ineffective at reducing symptoms of carpel tunnel, abdominal pain due to surgery and consistent, long-term general pain reduction (Murray, 1995; Chou et al., 2008; Reinhold et al., 2008; Plank and Goodard, 2009; Cho et al., 2012). Regarding psychopathology, acupuncture has demonstrated promising results for alleviating depression, bipolar disorder and mood associated with premenstrual disorder (Zhong et al., 2008; Dennehy et al., 2009; Zhang et al., 2010; Armour et al., 2018). Two meta-analyses demonstrated that acupuncture effectively treats major depressive disorder (MDD) and post-stroke depression (PSD) and should be considered an alternative treatment to antidepressants. Zhong and colleague’s meta-analysis (2008) found that acupuncture was as effective as the antidepressant fluoxetine. Acupuncture seems to be a safe and cost-effective form of treatment for MDD, PSD and bipolar disorder but the mechanisms by which acupuncture is effective for some—either through physiological, psychological or emotional alterations—is still debatable and more research is needed to unravel its authentic effects.

One issue with acupuncture is the placebo effect and a few studies have investigated this utilizing neuroimaging. Evidence from these studies show that expectations during acupuncture or sham (placebo control) has a physiological effect on the brain network, the same network that mediates a non-specific clinical response to acupuncture (Middlekauff et al., 2001; Qin et al., 2008; Harris et al., 2009). Bai and Lao (2013) suggest that divergent neural mechanisms exist and may possibly mediate specific aspects of acupuncture effects compared to placebo effects. Consequently, because a placebo effect or psychological expectation seems to play a role in the effectiveness of acupuncture, research design and control groups pose a challenge in researching authentic benefits. Specifically, the challenge is two-fold: does the mode of stimulation or the location of the acupuncture point bear physiological/psychological effects? Further research that has control groups with less confounding variables are needed to clarify the effectiveness of acupuncture and fine-tune the populations or conditions that benefit most from this ancient therapy.

### Reflexology: Tactile, Other-Administered

Reflexology requires applied pressure and manipulation of soft tissue, involving stimulation of reflex points on the feet and hands which are thought to correspond somatotopically to specific areas and organs of the body (Stephenson et al., 2007; Miller et al., 2013). Unlike acupuncture, there is less empirical evidence for the benefits and effectiveness of reflexology as an intervention. In 2007, a Cochrane review on reflexology did not find sufficient evidence to support its use and subsequent investigations after the review show conflicting evidence for its effectiveness as an alternative therapy (Wang et al., 2008; Miller et al., 2013). Some studies show that reflexology significantly decreased pain in cancer patients and women experiencing postmenstrual syndrome (Stephenson et al., 2000) and other studies show that reflexology does not have lasting effects on pain reduction (post 3 h stimulation) (Oleson and Flocco, 1993; Stephenson et al., 2003). Another study utilized the cancer patient’s partner as the reflexology administer and found that cancer patients reported a significant decrease in pain intensity and anxiety (Stephenson et al., 2007). Cancer patients reported a reduction in nausea, vomiting and fatigue after reflexology treatments (Yang, 2005). A more recent study found that for psychological factors, reflexology may effectively reduce stress in pregnant women (McCullough et al., 2018). Thus, reflexology is seen as a supplemental or self-care intervention that could relieve symptoms of pain, anxiety and/or stress but conflicting evidence exists for its authentic therapeutic effectiveness and few suggest that reflexology carries long-term healing properties or should replace medical intervention (Miller et al., 2013).

### Rolfing: Tactile, Other-Administered

Rolfing involves the myofascial structural integration or manipulation of muscle and soft tissue with the goal of loosening fascia layers, reposition muscles and aid body alignment (Hansen et al., 2014). Few studies have investigated the therapeutic effectiveness of Rolfing. Of these few, Weinberg and Hunt (1979) found that Rolfing helped reduce anxiety and suggests that Rolfing “releases” stored emotional tension in soft tissue. Hansen and colleague studied the effects of Rolfing on children (*n* = 2) with spastic cerebral palsy and found improvement in cadence and support time after 3 months of treatment.

Due to the scarcity of research and small sample sizes, more research is needed to investigate the effects of fascia and muscular manipulation. Some suggested hindrances to investigating Rolfing stem from a lack of congruence between the scientific and practitioner communities (Grimm, 2007). In order to further research investigating the effectiveness of Rolfing, efforts to bridge communication and cooperation between both fields is needed.

## Movement Therapies

### Dance/Movement Therapy: Tactile and Non-tactile, Other-Administered

Dance/movement therapy is one of the creative art therapies (music, art, poetry and drama therapy included) that uses movement and dance in a psychotherapeutic context, utilizing motion and emotion as a vehicle through which a person can gain a clearer definition of self (Payne, 2003). Dance/movement therapy originated in the United States in the 1940’s as a mind-body medical modality. Dance/movement therapy traces its roots to ancient forms of therapy that were utilized for healing, fertility enhancement, birth, and rituals involving sickness and death (Ritter and Low, 1996). Non-invasive and cost-effective, dance/movement therapy is used as an intervention for people with an array of emotional, cognitive and physical challenges and previous history of trauma (Serlin, 2007).

In the last 50 years, most research assessing dance therapy as an effective intervention focused on qualitative descriptions and case studies, asking questions about general well-being and subjective experience. The reason for an emphasis on qualitative rather than quantitative research was attributed to the nature of the creative arts therapies, which underscores creativity and subjective ways of knowing (Koch et al., 2014).

Research shows that dance/movement therapy affects mental, emotional and physical health by means of facilitating vestibular coordination, decreasing depressive symptoms (both in clinical and subclinical populations), enhancing psychological well-being in survivors of childhood sexual abuse, reduces anxiety, facilitates heart health in chronic heart failure patients and improves overall well-being in cancer patients (Couper, 1981; Lesté and Rust, 1990; Mills and Daniluk, 2002; Koch et al., 2007; Kiepe et al., 2012; Neto et al., 2014).

Jeong et al. (2005) propose that one possible mechanism by which dance/movement therapy is an effective form of treatment is in its ability to stabilize the sympathetic nervous system. The group measured plasma serotonin and dopamine concentrations pre and post dance/movement therapy in depressed adolescents and found an increase in serotonin and decrease in dopamine, suggesting that dance therapy ameliorates depressive symptoms by means of sympathetic influence.

Others suggest dance/movement therapy works to ameliorate negative symptomology, specifically those induced from previous traumatic experiences, through the therapeutic process of integrating emotions, cognitions and movement. Pierce (2014) highlights the imperative stages of effective dance/movement therapy: the first stage incorporates establishing trust between the client and dance therapist facilitating a sense of safety and stability, the second stage involves integration of previous traumatic events and the third stage is oriented toward rehabilitation and building the relational self through the context of other dance/movement therapy group members.

Several studies have demonstrated that dance/movement therapy is particularly effective at enhancing positive mood, well-being and body image while mending negative symptomology such as depression, anxiety and loneliness (Koch et al., 2014; Ho et al., 2018; Lange et al., 2018).

### Emotional Freedom Technique: Tactile, Self-Administered

Callahan (1985) originally developed a technique for self-tapping on acupuncture points as a means to decrease unwanted emotions or stress and Craig and Fowlie (1995) later simplified this method calling it the Emotional Freedom Technique (EFT) (Lint et al., 2006). This newer method involves concentrating on a specific psychological issue while simultaneously self-tapping on specified meridians on the body.

More novel than the aforementioned therapies, EFT significantly reduced symptoms in patients with PTSD. Church et al. (2013) found 90% of PTSD veterans no longer met PTSD clinical criteria with sustained effects at 3 (86%) and 6 months (80%) follow up. These findings are consistent with other studies that show positive long-term effects of EFT for trauma victims or PTSD patients (Church et al., 2009; Church, 2010).

While the mechanisms behind EFT are unknown, one study suggests that acupuncture meridians are not essential in therapeutic effectiveness. Waite and Holder (2003) found that tapping on parts of the body other than meridian points also elicited effects similar to those found in previous EFT studies. However, a more recent study found epigenetic changes may serve as the mechanisms by which EFT effectively reduces PTSD symptoms (Church et al., 2018). While more research is needed to extrapolate EFT’s treatment effects and the mechanisms by which it alters symptoms, one cogent advantage of EFT is ease of administration. Intrinsically a self-administered therapy, EFT is versatile and can be self-administered in a variety of settings. This aspect of EFT must be considered when examining its effectiveness with psychological disorders. More studies are needed to investigate EFT’s effectiveness upon psychopathological conditions other than stress, anxiety and trauma.

### Tai-Chi: Non-tactile, Self-Administered

Originating from China, Tai-Chi is a combination of meditative movements and martial arts that utilizes slowly performed, dance-like postures and movements. Tai-Chi also integrates relaxation of muscles and breathing and incorporates mental concentration.

A meta-analysis investigating tai-chi with elderly patients found beneficial effects on measures of general psychological well-being, depression, anxiety, general stress management and exercise self-efficacy (Chou et al., 2004). Their meta-analysis included three random control trials that used depression as an outcome measure (ES = −5.97; 95% CI −7.06 to −4.87), with *I*^2^ = 0%). Other studies further support tai-chi and its effectiveness at reducing anxiety and stress (Wang et al., 2004; Taylor-Piliae et al., 2006).

Tai-chi is also effective in promoting psychological well-being across cultures, including Western and Eastern samples. Wang et al. (2010) examined quantitative effect sizes for tai-chi on several measures of psychological functioning and found moderate to large effects sizes for reduction of stress (effect size [ES], 0.66; 95% confidence interval [CI], 0.23 to 1.09), anxiety (ES, 0.66; 95% CI, 0.29 to 1.03), and depression (ES, 0.56; 95% CI, 0.31 to 0.80), and enhanced mood (ES, 0.45; 95% CI, 0.20 to 0.69).

#### Mechanisms

Several possible mechanisms have been suggested as neurobiological contributors to the effectiveness of tai-chi in reducing symptomatology. Because tai-chi invokes physical relaxation, changes in brain waves (Field et al., 2013), decreased systolic and diastolic blood pressure and reduced cholesterol (Ko et al., 2006; Wolf et al., 2006) are suggested. These changes to autonomic and endocrine functioning by means of tai-chi through the combination of relaxation, movement, mental concentration and agility are also influenced by physical and mental training.

In summary, tai-chi is an effective body-centered therapy at reducing the psychopathological conditions of depression and anxiety. More research is needed explore the effects of tai-chi on other conditions and the mechanisms by which tai-chi is operative.

### Yoga: Non-tactile, Self-Administered

Yoga is a body-centered intervention designed to foster balance and health to the physical, mental, emotional and spiritual aspects of the individual. This body-centered intervention takes into account both physical postures, breath control, control of the senses, concentration and meditation (Ross and Thomas, 2010).

Yoga has been utilized as an alternative or complementary therapy for a variety of conditions and there exist conflicting evidence for its effectiveness at improving mental and physical health. Some populations seems to benefit from yoga, such as patients with chronic pain (Desveaux et al., 2015). A Cochrane review (Broderick et al., 2015) that included 8 studies found that yoga benefited individuals with schizophrenia on several mental health metrics including mental states (positive and negative syndrome scale), social functioning and overall quality of life but cautions results based upon low to moderate quality of evidence. They suggest there was insufficient evidence to consider yoga as superior to standard care for schizophrenics. Other Cochrane reviews support these findings regarding the limited strength of yoga in treating schizophrenia compared to standard care (Broderick and Vancampfort, 2017; Broderick et al., 2017).

Other populations seem to experience benefits that are similar to exercise alone, such as patients with bipolar disorder or lower back pain (Sherman et al., 2010; Cramer et al., 2013; Uebelacker et al., 2014). Buffart et al. (2012) found that cancer patients reported a decrease in stress and anxiety post yoga treatments and only a moderate change in fatigue, quality of life, and social and emotional functioning. Thus, both the population and disease condition in which yoga is applied seems to be a fundamental factor in determining effectiveness. More research is needed to examine those populations that seem to benefit most from yoga and more studies are needed to ascertain when alternative treatments such as exercise or meditation alone are equally as effective.

One possible factor that might explain the variability of yoga’s effectiveness is the component of meditation. As seen in patients with schizophrenia, mediation can catalyze psychotic experiences by inducing hyper-awareness of one’s internal state (Sethi and Bhargava, 2003; Kuijpers et al., 2007). Thus, utilizing yoga as an effective and beneficial treatment for differing conditions requires the assessment of both physical exercise and meditation upon that condition.

### Basic Body Awareness Therapy: Tactile, Other Administered

Basic Body Awareness Therapy (BBAT) is a form of body physiotherapy that aims to normalize body posture and increase overall balance and awareness of the body, both in relation to others and one’s environment. Common in Nordic countries, BBAT enhances awareness of one’s movement with the overall aim of integrating and facilitating bodily control by means of grounding/breathing exercises, massage and movement (Gard, 2005; Eriksson et al., 2007). There exists discrepancy within the literature regarding the usage and definition of either Body Awareness Therapy (BAT) or BBAT and its administration in either an individual or group setting such as Basic Body Awareness Group Therapy (BAGT). More precise terminology is needed to differentiate between these two psychosomatic physiotherapeutic treatments. However, there does exist two types of metrics which assess the effectiveness of BAT: the body awareness scale and the resource oriented body examination which measure body posture, muscle tension, respiration and heart palpitations (Eriksson et al., 2007).

BBAT has been effective at reducing physiological conditions such as fibromyalgia, chronic pain, irritable bowel syndrome and non-specific musculoskeletal problems (Gard, 2005; Eriksson et al., 2007) and more recent studies demonstrate potential mood altering effectiveness as well. One study found that BBAT improved major depressive symptoms (Danielsson et al., 2014) and others studies have investigated its effectiveness at reducing schizophrenic and anorexia nervosa symptoms (Hedlund and Gyllensten, 2010; Catalan-Matamoros et al., 2011). Two studies have investigated the use of BBAT in psychiatric clinics with patients who experienced mood, stress-related somatoform, behavioral and personality disorders. Both investigations found a significant decrease in symptoms and reduction in overall use and cost of psychiatric services (Gyllensten et al., 2003, 2009).

Thus, the administration of BBAT in psychiatric outpatient clinics seems to be the most promising line of research and more investigation is needed to evaluate its effectiveness within specific subpopulations. Further, more clarification is needed within the literature to not only define BAT or BBAT but evaluate the most effective form of BBAT as either a group or individual therapy.

## Conclusion

This review highlights a number of different body-centered therapies, namely interventions that have generated a substantial amount of research, and their effectiveness at reducing psychopathological conditions. There are additional body-centered interventions that seem to be gaining popularity that have yet to be investigated including hakomi, concentrative movement therapy, biosynthesis therapy, and character analytic vegetotherapy. However, of the interventions reviewed here, the most robust evidence was found for massage therapy because the higher number of meta-analyses and Cochrane reviews available (Moyer et al., 2004; Field, 2010, 2014, 2016; Hillier et al., 2010; Miozzo et al., 2016). Converging evidence from these studies and meta-analyses indicate massage was an effective intervention across age, including infants—premature and full-term—and elderly patients and effectively reduces varying psychopathological symptoms. Body-centered therapies with the least amount of evidence were Rolfing and reflexology. Studies examining these interventions had smaller sample sizes and no meta-analyses were available.

**FIGURE 2:**
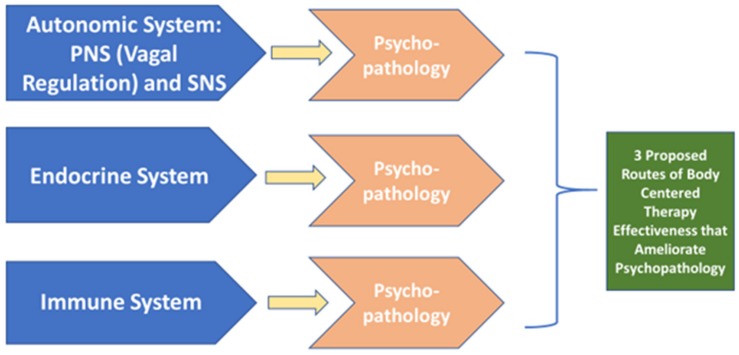
Possible routes by which body-centered therapies operate to ameliorate psychopathology.

Taken together, the evidence provided here indicates that certain body-centered therapies are effective at reducing certain psychopathological conditions (please refer to Table 1). Because there is converging evidence from several different therapies that body-centered interventions can reduce psychopathological conditions, there is evidence that suggests the body can communicate/influence the brain and alter psychopathological states. This suggests that alterations could be made to the brain and emotions by means of the body, providing some support that body-centered interventions act as effective interventions. Again, three possible routes are suggested as possible mechanisms by which the brain and body communicate: the autonomic system, the endocrine system and the immune system. We propose that if a body-centered intervention operates via one of these three routes and the targeted condition exists within these limits, a desirable health benefit could result. However, if both the targeted condition and body-centered intervention do not utilize one of these routes, then the therapy is theorized to yield ineffective results. Please see Figure 2.

**TABLE 1:** Taxonomy of body-centered interventions.

**Therapy**	**Administration type**	**Tactile form**	**Symptoms influenced**	**Evidence**
**Movement**				
Dance	Self	Both tactile and non-tactile	Enhances well-being and positive emotions, reduces negative emotions	Couper, 1981; Lesté and Rust, 1990; Mills and Daniluk, 2002; Koch et al., 2007; Kiepe et al., 2012; Neto et al., 2014

Emotional Freedom Technique	Self	Tactile	Decreases PTSD symptoms, anxiety, fear, stress	Church et al., 2009; Church, 2010

Yoga	Self	non-tactile	chronic pain reduction, increase physical health, potential to reduce stress and anxiety	Desveaux et al., 2015

Tai-Chi	Self	Non-tactile	Depression, increases general well-being	Chou et al., 2004; Field et al., 2013

Basic Body Awareness Therapy	Other	Tactile	Decrease in mood, stress-induced somatoform disorders, behavioral and personality disorders; schizophrenia; major depression and anorexia nervosa	Gyllensten et al., 2003, 2009; Hedlund and Gyllensten, 2010; Catalan-Matamoros et al., 2011; Danielsson et al., 2014

**Non-movement**				
Acupuncture	Other	Tactile	Reduces tension headaches/migraines, Parkinson’s disease symptoms, fibromyalgia, chronic neck and back pain, osteoarthritis pain	Middlekauff et al., 2001; Qin et al., 2008; Harris et al., 2009

Functional Relaxation	Self	Non-tactile	Reduces non-specific chest pain, asthma, and tension headaches	Loew et al., 2000, 2001; Lahmann et al., 2008a, 2009

Massage	Other and Self	Tactile	Increased weight and growth in pre-term infants, decreased cortisol and dopamine, increased serotonin, increased positive mood, decreased negative emotions, decrease in stress, increase in immunological response, increase in vagal tone	Field et al., 2005, 2010b; Field, 2010

Reflexology	Other and Self	Tactile	Decreases anxiety and stress	Wang et al., 2008; Miller et al., 2013

Rolfing	Other	Tactile	Decreases anxiety, improved gait in children with spastic cerebral palsy	Weinberg and Hunt, 1979; Hansen et al., 2014

We speculate that of the body-centered interventions mentioned that are found to be effective, the administration type seems to be critical. Self-administered therapies such as EFT, yoga, and dance therapy seem to hold two added variables that may contribute to their effectiveness: ease of administration (self) and focus of cognitive training and attention. We suggest that these additional factors may contribute to underlying mechanisms involved. Through cognitive training and attention, a possible fine-tuning of emotional regulation might be taking place.

In addition, evidence from body-centered interventions addresses the relationship between the body and the self. When the body and self are properly connected and integrated, healthy physiology and psychological functioning results. However, a rift between the body and the self creates a lack of integration and subsequently, inadequate communication between the two. The result is lack of control over the body or self, yielding pathological responses rather than proper functioning. In light of the body-self relationship, this generates more questions regarding the effectiveness of self versus other administered therapies and more research is needed to evaluate this.

### Clinical Application: Integrative Methodology and Toolbox Approach

Evidence from the abovementioned interventions indicates that certain populations can benefit from body-centered interventions as complimentary therapies. Due to the large number of individuals who already seek alternative therapies while undergoing traditional medical intervention, a novel treatment paradigm is needed. In addition to more research investigating the specific effectiveness of body-centered interventions, we suggest an integrative approach toward clinical intervention. This model emphasizes an interdisciplinary methodology in which physicians and therapists work together to generate a treatment plan for an individual. Rather than compartmentalizing symptoms and conditions, a dialogue between professionals is needed in which they collaboratively craft a treatment plan that incorporates body-centered interventions.

In order to properly tailor the treatment plan to the individual, we suggest considering the array of body-centered interventions as a toolbox of interventions. Rather than viewing body-centered interventions as a whole, each therapy should be considered in terms of desired effects, symptomology and the individual’s history. If one particular body-centered intervention does not generate desired results, a different therapy could be implemented. Also, the toolbox of body-centered interventions can be viewed as progressive: patients can move from less intensive to more intensive therapies. An example of this would be a patient with a history of physical/sexual abuse and a progressive treatment plan of (1) EFT, then once comfortable, moving to (2) reflexology and finally, (3) full body massage.

Further, as demonstrated herein, these interventions are currently recognized as alternative or complementary interventions. As empirical evidence grows and clarifies which interventions are effective or not and which populations benefit from such treatments, we propose that these interventions move from being seen as “alternative” to more primary in the construction of an individual’s medical treatment plan. For a clinical example of the effectiveness of a body-centered intervention, please see Appendix A.

In conclusion, body-centered therapies are a promising line of intervention that augments the role of the body within the bidirectional communication pathway between the brain and body. These interventions alleviate physical and emotional symptoms via the manipulation of the body, which, in turn, supports the body-self connection. Further research is needed to disentangle the mechanisms underlying body-centered interventions and consider an integrative approach that includes body-centered interventions in treatment plans for psychological disorders.

## Author Contributions

MT investigated empirical evidence, organized and wrote the manuscript. SP provided theoretical framing. ST provided clinical vignette.

## Conflict of Interest

ST was employed by Dancing Dialogues. The remaining authors declare that the research was conducted in the absence of any commercial or financial relationships that could be construed as a potential conflict of interest.

## Appendix A

### Clinical Vignette

Identifying features have been altered to maintain confidentiality.

An Application of Dance/Movement Therapy Integration of Somatic and Cognitive Experience in the Therapeutic Process

Jerry is a 35 years old female, tall and of average weight, who works as an executive in a large business and typically enjoys an active physical life. She began experiencing pain and a restricted range of motion in her right shoulder, procured medical attention and received a diagnosis of frozen shoulder. Following medical advice, she undergoes physical therapy for 4 months and experiences no improvement. Her primary physician refers her to dance/movement therapy.

At the first therapy session, Jerry briskly enters the office holding her right arm tightly across her chest with the palm of her hand snuggled next to her clavicle. She cautiously sits down, bracing her upper torso and holding her body upright away from the back of the chair. The flow of Jerry’s breath was short and shallow, seemingly contained in her upper torso.

Jerry describes her situation as follows;

“*I don’t understand what has happened to me. My life has always revolved around physical activities and pushing myself to do my best. I grew up in a family that was all about achievement. Everyone in my family works really hard and we all succeed. I learned early on that being physical has been a core way that I balance my emotional and work life. I have not been able to do anything these four months and I’m depressed and discouraged.”*

The goal of the dance/movement therapy was to help Jerry become aware of the interconnections among her bodily sensations, personal movement style, emotional reactions and thoughts that create an embodied sense of herself. During the initial stage of treatment, Jerry learned to identify how she holds her breath when experiencing stress and what aspects of her life are most distressful. First, Jerry was asked to deepen her breath in vertical, sagittal and horizontal directions that promotes calmness while listening to gentle flowing harp music. As her torso became more relaxed, she begins to recall that she learned to “*hold myself so erect and tightly as a child, trying to keep up with all my family’s expectations of achievement.*”

Then, accompanied by music with a strong beat, Jerry was asked to explore an erect posture, while holding her right arm across her chest and listening to music with a strong beat. During this movement improvisation, Jerry spontaneously stretched her right arm out in front of her, extending her elbow and making a pulsing gesture with her hand, pushing away from her torso. She suddenly stopped, drawing her arm back across her chest, her hand by her heart, hollowing her upper torso. She began to cry. The therapist changed back to the harp music and supported her, enabling her to connect to the flow of her breath.

In this moment, her body, emotions and thoughts were connected and integrated, allowing Jerry to recall the feeling of being overwhelmed as a child, and needing to protect her heart from family pressure. She yearned for affection when feeling overwhelmed, but her achievement-oriented parents viewed this need as a weakness. By exploring and identifying the metaphors associated with her frozen shoulder, Jerry was able to be kinder to herself, and ask for physical affection from her spouse when stressed. Her frozen shoulder improved and she was able to resume an active, balanced life.
